# Cardiorespiratory fitness mediates the effects of aging on cerebral blood flow

**DOI:** 10.3389/fnagi.2014.00059

**Published:** 2014-04-07

**Authors:** Benjamin Zimmerman, Bradley P. Sutton, Kathy A. Low, Mark A. Fletcher, Chin Hong Tan, Nils Schneider-Garces, Yanfen Li, Cheng Ouyang, Edward L. Maclin, Gabriele Gratton, Monica Fabiani

**Affiliations:** ^1^Neuroscience Program, Beckman Institute for Advanced Science and Technology, University of Illinois at Urbana-ChampaignUrbana, IL, USA; ^2^Department of Bioengineering, Beckman Institute for Advanced Science and Technology, University of Illinois at Urbana-ChampaignUrbana, IL, USA; ^3^Beckman Institute for Advanced Science and Technology, University of Illinois at Urbana-ChampaignUrbana, IL, USA; ^4^Department of Psychology, Beckman Institute for Advanced Science and Technology, University of Illinois at Urbana-ChampaignUrbana, IL, USA

**Keywords:** cardiorespiratory fitness, aging, arterial spin labeling, cerebral blood flow, vascular health, perfusion

## Abstract

The brain's vasculature is likely to be subjected to the same age-related physiological and anatomical changes affecting the rest of the cardiovascular system. Since aerobic fitness is known to alleviate both cognitive and volumetric losses in the brain, it is important to investigate some of the possible mechanisms underlying these beneficial changes. Here we investigated the role that estimated cardiorespiratory fitness (eCRF) plays in determining the relationship between aging and cerebral blood flow (CBF) in a group of older adults (ages 55–85). Using arterial spin labeling to quantify CBF, we found that blood flow in the gray matter was positively correlated with eCRF and negatively correlated with age. Subsequent analyses revealed that eCRF fully mediated the effects of age on CBF in the gray matter, but not in the white matter. Additionally, regional measures of CBF were related to regional measures of brain volume. These findings provide evidence that age-related effects on cerebrovascular health and perfusion in older adults are largely influenced by their eCRF levels.

## Introduction

Normal aging is accompanied by structural and functional changes that occur throughout all bodily systems, which in turn lead to physiological and psychological changes. These complex and interacting biological changes are at the basis of many of the diseases prevalent in older adults and also contribute to age-related decline in cognitive function. It is no surprise that recent evidence has begun to uncover links between the health of the central nervous system, and consequently cognition, and the health of the cardiovascular system in aging populations. For example, Chao et al. (2010) recently demonstrated that baseline measures of brain perfusion using arterial spin labeling (ASL) are predictive of cognitive decline and progression to dementia in older adults.

The cerebral vasculature is subject to the same age-related changes that affect other parts of the cardiovascular system, including compromised endothelial function and arterial stiffening (Izzo, 2005). In fact, there is ample evidence that the health and functioning of the brain's vasculature decreases with age. For example, vascular reactivity and compensatory vasodilation become impaired with age (Safonova et al., 2004; see also Fabiani et al., 2014). Furthermore, mean cerebral blood flow (CBF) declines steadily across the lifespan, with gray-matter blood flow significantly decreasing with age, as shown by Parkes et al. (2004), although this study did not investigate adults older than 67. Decline in CBF is related to an overall decline in brain volume, but research has shown that the decline in CBF is more than just a reflection of these volumetric changes (Chen et al., 2011). Thus, CBF may provide a good general index of changes in the health of the cerebral vasculature, in the same way that ventricular expansion has been used as an index of overall tissue shrinkage in the brain (e.g., Raz and Rodrigue, 2006), since it may reflect the overall state of perfusion and the rate of angiogenesis in the brain.

Fortunately, it may be possible to alleviate the decline in vascular health that accompanies aging. There is evidence that aerobic exercise is associated with improved endothelial function, greater arterial elasticity, and reduced risks for vascular diseases in aging adults (Clarkson et al., 1999; DeSouza et al., 2000). Likewise, animal studies have shown improvements in endothelial function and increases in CBF with increased aerobic exercise (Endres et al., 2003; Gertz et al., 2006). These findings become especially important when cognitive health—the degree to which cognitive functionality is maintained across the lifespan—is considered. Research suggests that increased aerobic fitness can prevent or even reverse cognitive decline (Hillman et al., 2008). For example, high-fit older adults have more preserved gray and white matter in the frontal, parietal, and temporal cortex than low-fit older adults (e.g., Colcombe et al., 2006; Gordon et al., 2008). These volumetric differences in high-fit older adults are accompanied by increased performance in cognitive tasks compared to age-matched low-fit adults (Kramer et al., 2006). It could be hypothesized that aerobic fitness affects the process of cognitive aging, at least in part, through its remediating effects on the cerebral vasculature and perfusion.

Given this evidence, it is important to determine to what extent age-related declines in CBF (used as an indicator of cerebrovascular health) are related to lower levels of fitness (which is an individual difference variable that could be subject to intervention). Previous research has shown that aerobically-trained males have a reduction in their age-related decrease in blood flow velocity in the middle cerebral artery measured using Doppler sonography (Ainslie et al., 2008). However, this method does not probe perfusion directly. Therefore, in the current study we used ASL, a non-invasive magnetic resonance (MR) technique that is capable of determining measures of CBF more directly (see Borogovac and Asllani, 2012 for a recent review on ASL).

ASL utilizes the intrinsic water molecules in the blood as a tracer by tagging them with a saturating or inverting radiofrequency (RF) pulse. As this “tracer” diffuses within brain tissue there is a reduction of tissue magnetization, and, as a consequence, a reduction in the MR signal. An image is taken during this process at some predetermined transit time. The process is repeated without labeling the water, and the difference between this control image and the tagged image yields a perfusion image. Using this method to derive CBF in aging adults, the present study investigated the relative contributions of age and estimated cardiorespiratory fitness (eCRF) on CBF in different regions of the brain, corresponding to areas that have been shown to decline most substantially, both anatomically and functionally, during the normal aging process.

## Methods

### Subjects

Fifty-five adults (aged 55–87), who satisfied the inclusion criteria listed below, were recruited from the Champaign-Urbana area. Subjects with serious or chronic medical conditions, a history of major neurological or psychiatric disease, or a history of drug abuse were excluded from this study. Additionally, subjects were screened and excluded if they showed signs of dementia and depression. Participants needed to score at least 51 on the modified Mini-Mental Status examination (Mayeux et al., 1981) and less than 14 on Beck's Depression Inventory (Beck et al., 1996) in order to participate in the study. Participants who smoked more than half a pack of cigarettes and/or consumed more than two drinks per day were also automatically excluded. All participants were right-handed as assessed by the Edinburgh Handedness Inventory (Oldfield, 1971), had normal or corrected-to-normal vision, were non-smokers, and were native speakers of English. Participants were also assessed for level of education, vocabulary (Shipley, 1940), and intelligence quotient (Kaufman and Kaufman, 2004) (Table 1). Participants' blood pressure was taken at three time points across the experiment and averaged to provide measures of systolic and diastolic blood pressure^1^. Pulse pressure was derived by taking the difference between systolic and diastolic blood pressure. All participants were paid for their time in the study and signed informed consent in accordance with the University of Illinois at Urbana-Champaign's Institutional Review Board.

**Table 1:** **Overall demographic characteristics**.

	**Mean**	***SD***	**Range**
Age (years)	69.15	8.31	55–85
eCRF (metabolic equivalents)	6.80	2.25	1.58–11.31
Systolic blood pressure	135.41	14.20	105.67–167.33
Diastolic blood pressure	79.40	7.62	63.33–96.00
Pulse pressure	56.01	13.58	34.33–91.33
Education (years—capped at 20)	16.87	2.89	12–20
Modified mini-mental status examination	55.27	1.30	51–57
Beck's depression index	3.22	3.55	0–14
Shipley's vocabulary test	36.17	2.47	31–40
Kaufman brief intelligence test	117.41	10.92	95–142

N = 41.

In all, data from 14 participants were discarded from subsequent analysis. Of these, five participants did not have a complete MRI dataset, three participants were excluded due to movement artifacts, and six participants were removed as outliers for having at least one MRI measure that was 3 standard deviations different from the mean. The final sample included 41 older adults (aged 55–85, mean age = 69.2 years, 22 females). 16 of those participants reported being on medication for their blood pressure. The demographic characteristics of this sample are presented in Table 1.

### MRI acquisition

All MRI data collection occurred at the Biomedical Imaging Center using a 3T Siemens (Erlangen, Germany) Trio scanner using a standard body coil transmission and a 12-channel head array receive coil.

Subjects were instructed to perform a breath-holding task according to a visual cue, which included six repetitions of alternating periods of breath-holding after expiration (18 s) and self-paced breathing (36 s) for a total acquisition time of 5 min and 24 s. Breath holding tasks can be used to induce vasodilation in the brain and thus increase CBF and allow for the investigation of vascular reactivity along with resting levels of perfusion. Instructions for the breath-holding experiment were presented using EPRIME (Psychology Software Tools, Pittsburgh) and displayed via back projection (BrainLogics, Psychology Software Tools, Pittsburgh). To minimize motion during the breath-holding period, padding was used to stabilize the subject's head.

Six axial imaging slices passing through the middle of the lateral ventricles and covering part of the frontal cortical areas were acquired with localized pseudo-continuous Transfer Insensitive Labeling Technique (pTILT) ASL sequence (Ouyang and Sutton, 2011), and the labeling slice was placed inferior to the imaging slab with a 10 mm gap. As part of a larger study not discussed here, near-infrared optical data were recorded concurrently from a patch on the forehead during the MR experiment. Therefore, the axial slices were lined up with the optical coverage. The pTILT ASL method is less sensitive to the transit time issues that may confound other ASL methods as a function of age. In fact, this method tags close to the slice of interest, minimizing the differences in transit time that may result from age-related changes in the vasculature.

The imaging and tagging parameters of the localized pTILT ASL sequence were: windowed-sinc 45° RF pulses with 2560 μ s-duration, tagging repetition spacing = 30 ms, number of concatenated RF pulse pairs = 100, tagging duration = 3 s, post-labeling delay = 0.5 s, tagging slice thickness = 10 mm, gradient spoiler duration and amplitude = 4000 μ s/[±10, ±12, ±14, ±16] mT/m, SE-EPI readout, FOV = 220 × 220 mm, scan matrix size = 64 × 64, *TR*/*TE* = 4500/44 ms, slice thickness = 6 mm, slice gap = 1.2 mm, 36 control and 36 tag repetitions, scan time of one acquisition = 5 min and 24 s.

To assist with the registration procedure, two additional brain scans were taken: a high-resolution 2D turbo-spin echo (TSE) acquisition with the imaging slices at the same location as the ASL images, and a high-resolution T1-weighted 3D anatomical image. The T1-weighted brain image was acquired using a 3D MPRAGE (Magnetization Prepared RApid Gradient Echo) protocol [*TR* = 1900 ms, *TI* (inversion time) = 900 ms, *TE* = 2.32 ms, field of view = 230 × 230 × 172.8 mm^3^ (sagittal), matrix size = 256 × 256 × 192, flip angle = 9°, slice thickness = 0.9 mm].

### MRI data processing

The pTILT functional data processing was carried out using SPM 8 (Wellcome Department of Cognitive Neurobiology, University College of London, UK) and FSL 4.1.4 (FMRIB Software Library; http://www.fmrib.ox.ac.uk/fsl). The fMRI modeling of the BOLD, baseline perfusion, and activation perfusion responses were determined using the general linear model (GLM) with the ASL modeling framework described by Hernandez-Garcia et al. (2010). The unsubtracted pTILT data were first realigned to remove motion artifacts. Four regressors were modeled in the GLM analysis: (1) the breath hold task BOLD response (a canonical hemodynamic response function, HRF); (2) baseline perfusion (a consistent, alternating waveform); (3) activation blood flow (an alternating waveform during the breath-hold task); and (4) a baseline signal (uniform intensity). After regression analysis, gray and white matter masks were formed from segmenting the T1 structural scan using FSL's FAST software (Zhang et al., 2001). The gray and white matter masks were then transformed into the subject's pTILT space using a registration between the control image in pTILT and the MPRAGE from FSL's FLIRT (Jenkinson and Smith, 2001).

#### Regional measures

**Figure 1:**
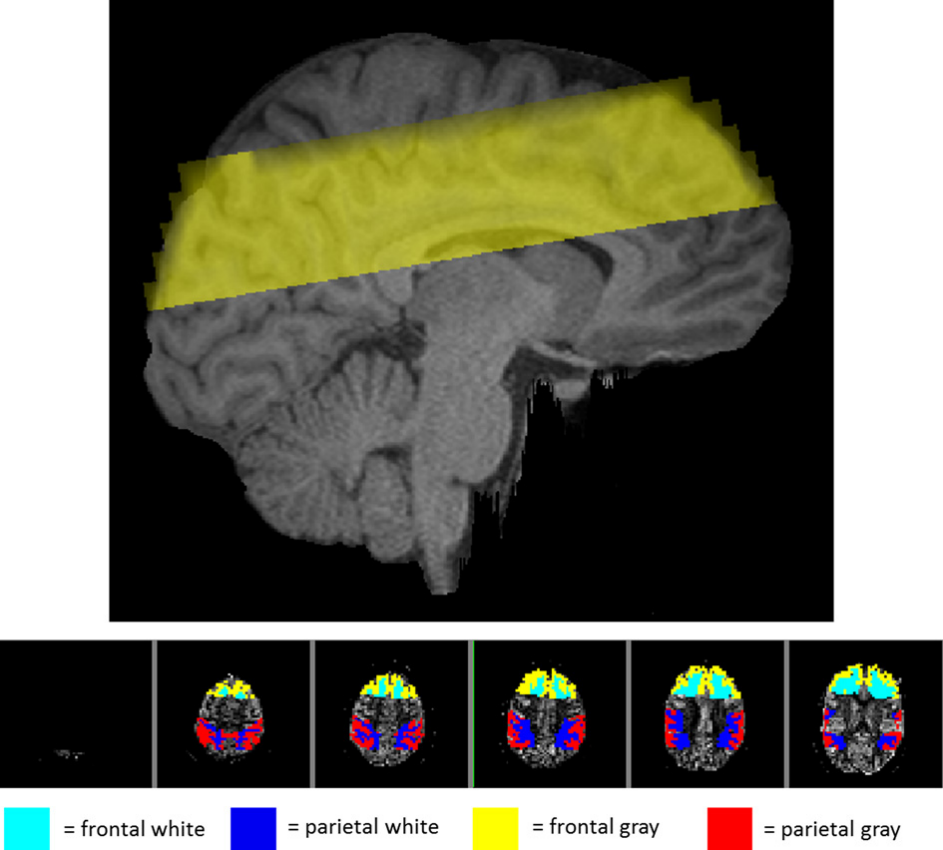
**View of the regions imaged in one representative subject. Top panel:** sagittal view of the location of the imaging volume taken in the study for one representative subject. **Bottom panel**: axial view of the imaging slices shown from superior (left) to inferior (right) with the frontal and parietal regions highlighted. The teal and blue areas represent the white matter in the frontal and parietal regions, respectively. The yellow and red represent gray matter in the frontal and parietal regions.

In addition to the global gray and white matter masks above, the Harvard-Oxford cortical and subcortical structural atlases provided by FSL were used to isolate activity in more localized areas of the brain. A linear registration between the subject's MPRAGE and the atlas was used to bring regions back into the individual subject's MPRAGE space and then on to the pTILT space. A frontal region was isolated by dilating the frontal pole with a 3 × 3 × 3 voxel kernel three times. The parietal region was isolated by combining five parietal areas including the postcentral gyrus, superior parietal lobule, supramarginal gyrus, anterior division, supramarginal gyrus, posterior division, and the angular gyrus. Averaging activity in only these regions provided separate regional measurements for frontal and parietal analysis. Figure 1 (top panel) shows a sagittal section with the imaging volume. Figure 1 (bottom panel) provides an example of the locations of the frontal and parietal regions for a single participant within the six slices imaged. Because the six axial slices were lined up with the optical recording patch on the forehead, the temporal lobes were not adequately covered for regional analysis.

#### Motion correction

In several subjects, significant head motion artifacts were observed at particular locations in the time series of the pTILT breath-holding data. In order to reduce the motion influence and increase the reliability of estimation from other time points, the SPM 8 Robust Weighted Least Square (rWLS) toolbox (Diedrichsen and Shadmehr, 2005) was used. The rWLS toolbox reveals images that are impacted by motion or other noise, based on the residual-mean-square estimate, which is calculated by adding up the squared residuals over the whole volume for each individual time point when applying the linear model. As an alternative to deleting data points that have been contaminated by motion, the rWLS toolbox “soft”-excludes those images by weighting each observation with the inverse of its variance. Since image volumes that have been corrupted by motion will have high variance relative to the linear model, the “soft”-exclusion method results in the “bad” images being significantly down-weighted in the subsequent analysis (Diedrichsen and Shadmehr, 2005). Nevertheless, the contrast between breath-holding and baseline measurements was invalidated by the present of consistent movements during the breath-hold period in some subjects. For this reason we focused instead on the baseline period.

#### CBF quantification

**Figure 2:**
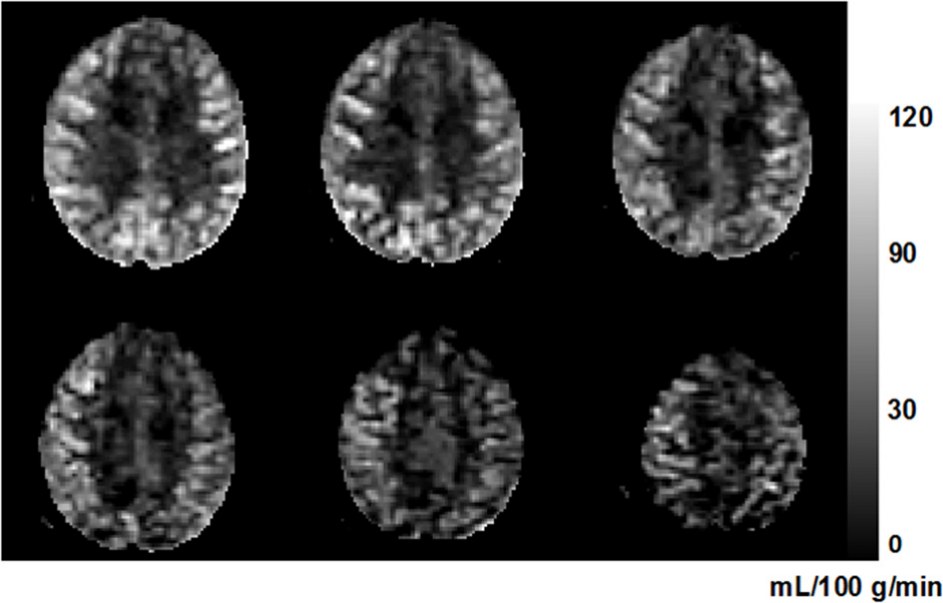
**An example perfusion image from a 63-year-old subject**. Six axial slices are shown from inferior (top left) to superior (bottom right). The higher perfusion in gray matter compared to that in white matter can be clearly seen. The unit of the gray-scale bar is mL/100 g/min.

Baseline perfusion images in mL/100 g/min units were calculated, based on a single compartment model in which no blood exchange is assumed (Ouyang and Sutton, 2011):

$$CBF=\frac{△M}{M_{0}}\cdot \frac{6000}{\lambda_{blood}\cdot T_{1,\;blood}}\cdot \text{exp}\left(\frac{w+T_{slc}\cdot \;\left(n-1\right)}{T_{1,\;blood}}\right)\cdot \text{ exp}\left(\frac{TE}{T_{2,\;blood}}\right)$$

where Δ*M* is the estimated coefficient of the tag-control difference (i.e., the perfusion-weighted control-tag image); *M*_0_ is the estimated coefficient of the static tissue signal (i.e., the control image); λ_*blood*_ is the water content of blood (0.9 as used in Chalela et al., 2000); *w* is the post-labeling delay (0.5 s); *T*_1, *blood*_ (1680 ms at 3 T) and *T*_2, *blood*_ (275 ms at 3T) are the longitudinal and transversal relaxation rates of blood (Stanisz et al., 2005); *T_slc_* is the acquisition duration of one single slice, and *n* is the index of acquired slice; and *TE* (44 ms) is the echo time of the SE-EPI sequence. Figure 2 shows an example of perfusion image in six axial slices from one subject.

### Anatomical volume estimation

Structural MRI images were processed with FreeSurfer (Dale et al., 1999). FreeSurfer allows for automated segmentation of cortical and sub-cortical volumes (Fischl et al., 2002, 2004a,b; Desikan et al., 2006). Estimates of cortical volumes were obtained using an automated probabilistic labeling procedure based on the Desikan-Killiany anatomical atlas (Fischl et al., 2002; Desikan et al., 2006). Subcortical and cortical volumes were normalized by intracranial volume using a co-variance approach to account for volumetric differences in head size (Jack et al., 1989; Buckner et al., 2004)^2^. FreeSurfer output was inspected for errors through extensive visual screening performed by three different highly trained individuals, with each person examining all slices for errors. Corrections to FreeSurfer output were performed according to the methods found online (http://surfer.nmr.mgh.harvard.edu/fswiki/FsTutorial/TroubleshootingData).

### Cardiorespiratory fitness

The “gold standard” measure for physical fitness is VO_2max_, which is a measure of the maximum oxygen consumption in an individual's body obtained during a maximal graded bout of exercise. However, older and low-fit individuals may have conditions that prevent them from participating in the stressful exercise routine. Since these individuals are of crucial interest for the examination of the relationship between CBF, fitness, and aging, in the current study cardiorespiratory fitness (eCRF) was estimated according to this equation (see Jurca et al., 2005):

eCRF = Gender (2.77) − Age (0.10) − Body Mass Index (0.17) − Resting Heart Rate (0.03) + Self-Reported Activity Score + 18.07.

This measure utilizes easily acquired parameters that are highly predictive of VO_2max_(Jurca et al., 2005). It has been demonstrated to approximate VO_2max_ with good accuracy in a large sample (*N* > 10,000), and recently, Mailey et al. (2010) extended the validity of the eCRF estimate specifically to older adults, ranging in ages from 60 to 80. McAuley et al. (2011) further validated this measure for smaller sample sizes by showing that there was no significant difference between this estimated measure of eCRF and the gold-standard, physician-supervised, maximal exercise test in a sample of 86 older adults. Finally, in a survey of over 32,000 individuals ranging in age from 35 to 70 years, Stamatakis et al. (2013) found eCRF to be a good predictor of cardiovascular (and overall) mortality, comparable to associations between exercise testing CRF and mortality.

### Analysis of eCRF

The current study was focused on the relationship between mean CBF, age, and eCRF. Since motion was correlated with the breath-holding task itself, even after using rWLS to correct for movement, we lacked sufficient power to analyze the activation CBF during the breath-holding intervals, and therefore focused on the baseline CBF, corresponding to rest periods.

Since we sought to determine the impact of eCRF independently of known gender differences in VO_2max_ (Hutchinson et al., 1991), we first regressed out gender from each of our variables of interest and then used the residuals for all subsequent comparisons. We used regression analysis to test the simple effects of age and eCRF on CBF for each region of the brain.

To test whether eCRF mediated the effects of age on CBF, we performed a mediation analysis (see Baron and Kenny, 1986) using multiple regression. This type of mediation analysis consists of three steps. The first two steps are aimed at demonstrating the significant relationships between the proposed mediator (eCRF) and the independent variable (age) and between the dependent variable (CBF) and the independent variable (age), which we knew to be true from our previous analysis of bivariate correlations. The final step involves regressing the dependent variable on both the independent variable and mediator. After this step, the unique effect of the independent variable when the mediator is included as a predictor variable is compared to the simple effect of the independent variable alone. If including both the mediator and independent variable into the regression equation eliminates CBF's dependence on the independent variable (age), then the remaining significant variable is said to fully mediate the effects of the other on the dependent variable (Baron and Kenny, 1986). Three separate mediation analyses were conducted, one examining global effects and the other two exploring mediation in parietal gray and white matter, as these were the cases in which we observed all of the prerequisite relationships.

Various formal statistical tests have been devised for the purpose of directly testing the significance of the mediating effect. One reason for this is that the rules that Baron and Kenny provide for determining whether a mediation exists do not account for the possibility of observing a loss in the significance of the relationship between the independent and dependent variables without a significant change in the size of the actual coefficient (Holmbeck, 2002). The Sobel test (Sobel, 1982) analyzes the significance of the change in the predictive ability of the independent variable on the dependent variable before and after a mediator variable is included in the analysis, and thus provides a more formal treatment of the mediation. However, because the Sobel test depends on a normally distributed mediation effect, more recent work has recommended the use of bootstrapping the sampling distribution of the mediation effect to build an empirically based confidence interval (Preacher and Hayes, 2004). In consideration of these recommendations, we report the confidence intervals established through Preacher and Hayes' bootstrapping procedure using 10,000 resamples.

## Results

Table 2 reports the means and standard deviations of the mean CBF measures by region, for gray and white matter separately. Dependent sample *t*-tests revealed that gray matter CBF was significantly greater than the white matter CBF globally [*t*_(38)_ = 22.52, *p* < 0.001], as well as in the frontal [*t*_(38)_ = 13.19, *p* < 0.001] and parietal [*t*_(38)_ = 21.71, *p* < 0.001] regions that we investigated.

**Table 2:**
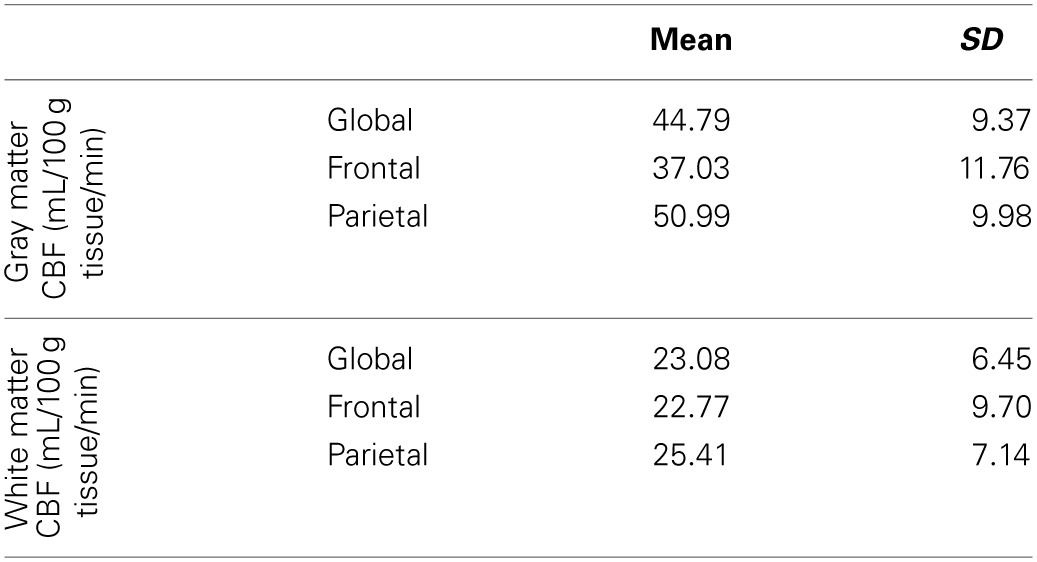
**Mean and standard deviation of global and regional blood flow measures**.

**Figure 3:**
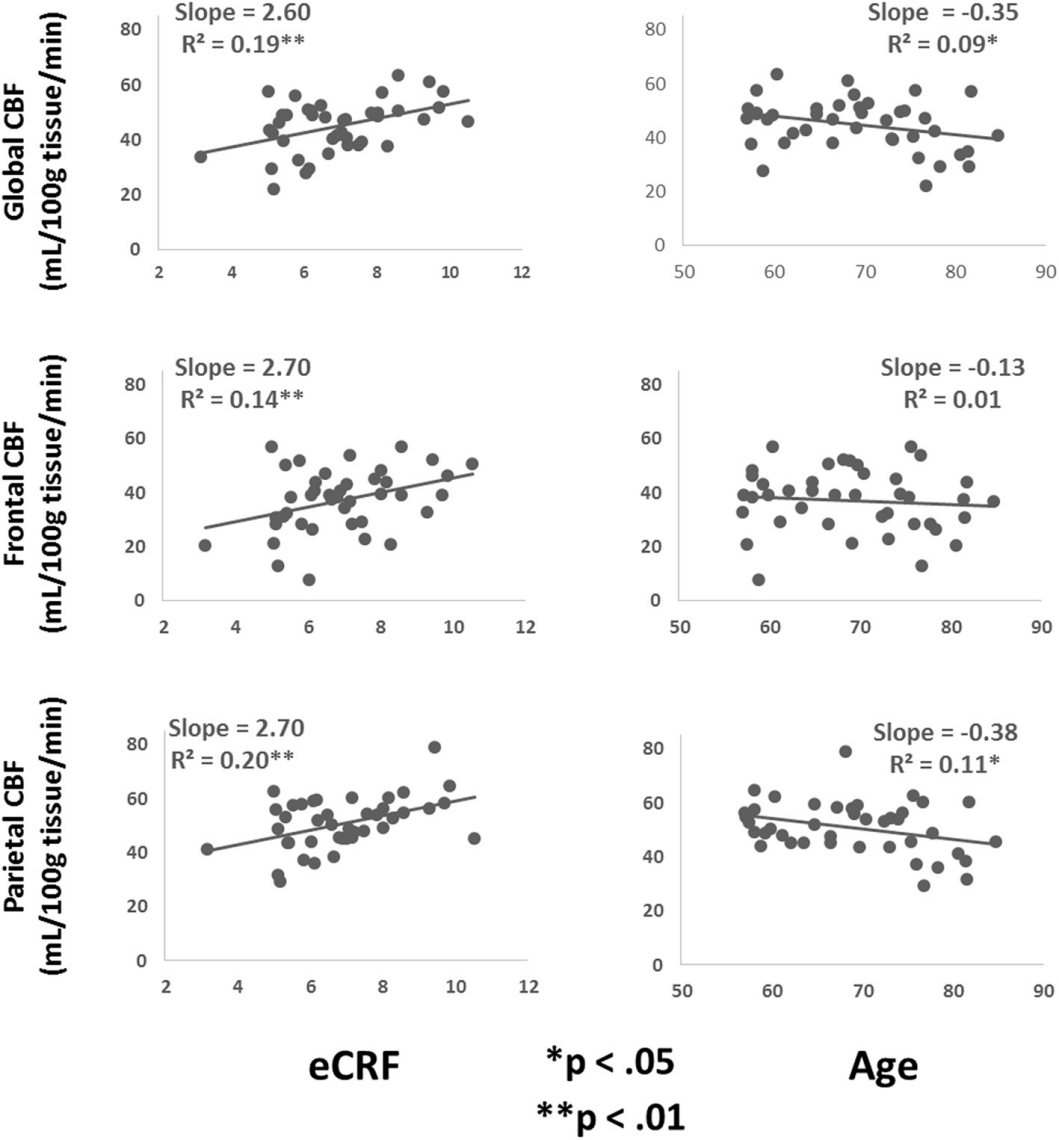
**Scatter plots depicting the relationships between CBF and eCRF (left column) and age (right column) in global (top), frontal (middle), and parietal (bottom) gray matter regions of interest**.

**Figure 4:**
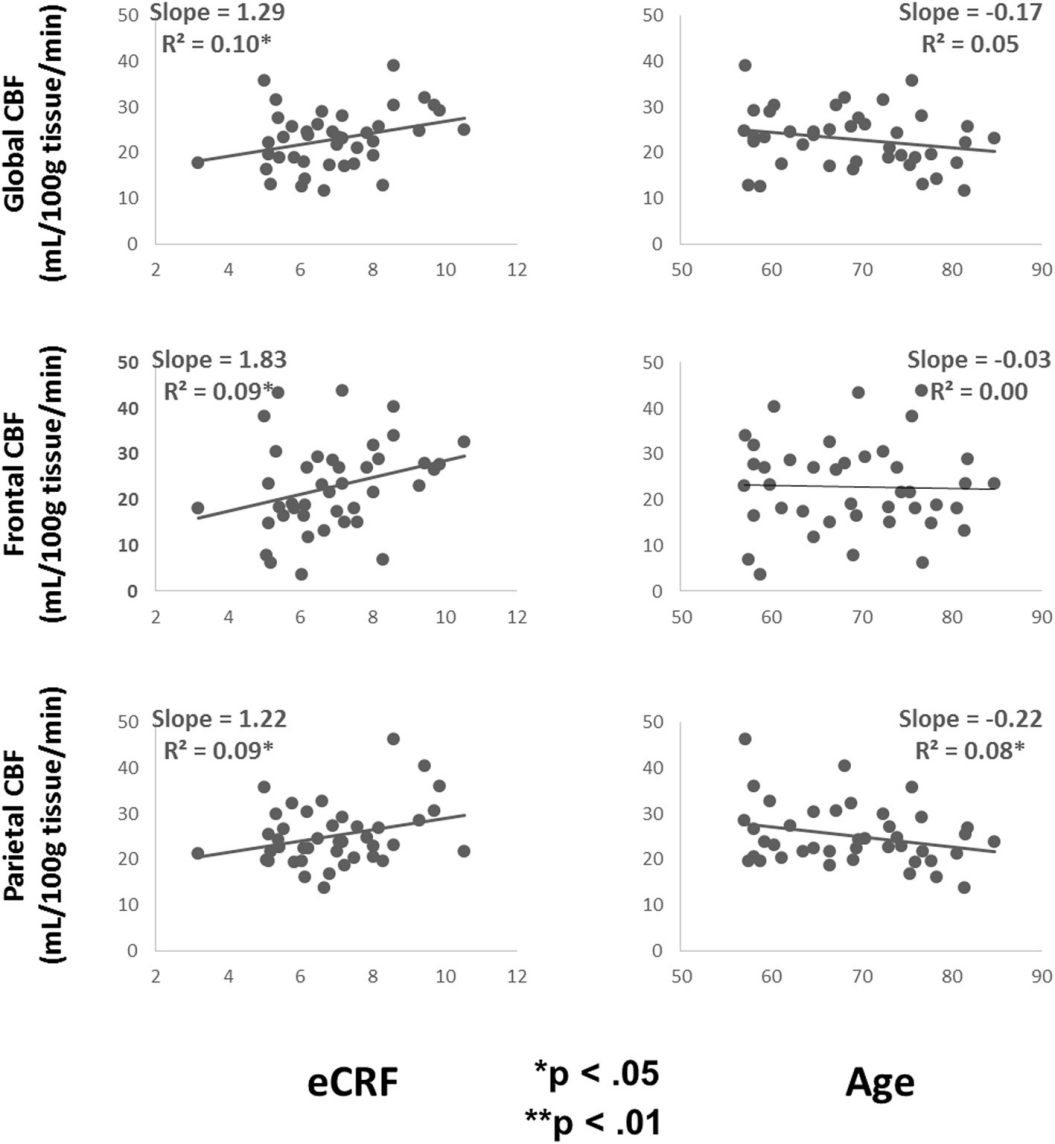
**Scatter plots depicting the relationships between CBF and eCRF (left column) and age (right column) in global (top), frontal (middle), and parietal (bottom) white matter regions of interest**.

Table 3 shows the partial correlations between the measures of CBF, age, eCRF, and blood pressure after controlling for gender. Based on previous research, we hypothesized that eCRF would be positively correlated with CBF whereas age would be negatively correlated with it, even in our restricted age-range sample of older adults. It should be noted here that there were no significant differences between participants who took medication to control their blood pressure and participants who did not on any of the measures reported in this paper. Given the previously established directionality of blood flow changes with age and our corresponding directional hypotheses, we performed one-tailed significance tests. Figures 3, 4 (top panels) show the relationship between the independent variables (eCRF and age) and gray and white matter mean flow, respectively. As we expected, eCRF positively correlated with gray matter mean flow, and age negatively correlated with gray matter mean flow. eCRF was also correlated with the white matter mean flow (Figure 3, top panel). However, age was only marginally predictive of white matter mean flow *r*_(38)_ = −0.22, *p* = 0.08.

**Table 3:**
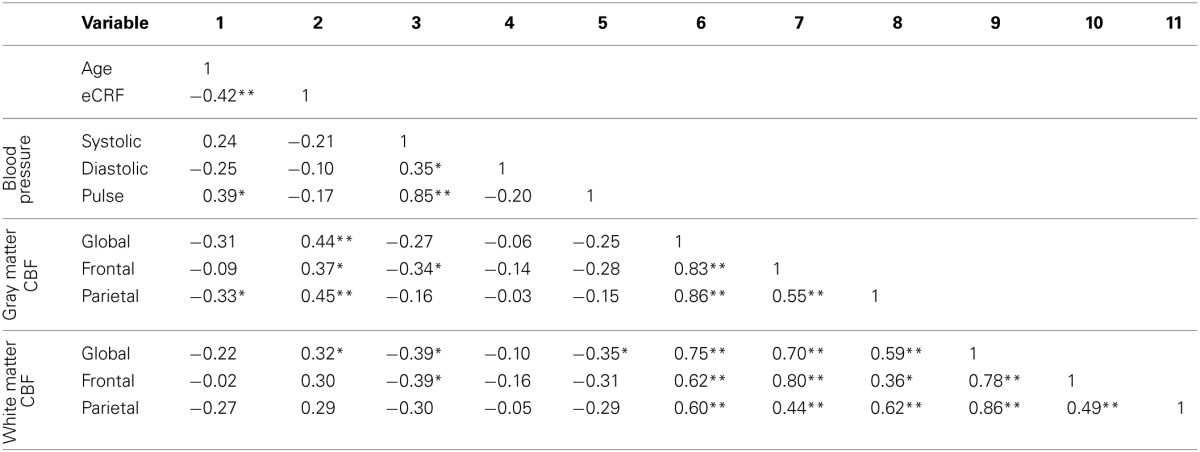
**Partial correlations between age, eCRF, blood pressure, and CBF, controlling for gender**.

Significance tests between groups (2-tailed): df = 38, ^*^p < 0.05, ^**^p < 0.01.

Because many age-related effects on cognition are related to attention control and working memory functions (see Fabiani, 2012), which involve the frontal and parietal regions of the cortex, and because other studies have shown more severe rates of brain deterioration with aging in these regions (Resnick et al., 2003; Raz and Rodrigue, 2006; Hillman et al., 2008), we examined how age and eCRF affected the mean blood flow in these specific regions of the brain using the same linear regression analysis. Figures 3, 4 (middle panels), respectively, show the relationships with frontal gray and white matter mean flow. In frontal regions, eCRF predicted gray and white matter mean flow, similarly to what we found when examining global effects. In contrast, frontal regions did not show a reliable relationship between age and mean CBF in either the gray, *r*_(38)_ = −0.09, *p* = 0.28, or white matter, *r*_(38)_ = −0.02, *p* = 0.44. Figures 3, 4 (bottom panels) show the correlations with parietal mean flow. In parietal regions, we observed relationships similar to those reported in the global analysis, with both age and eCRF predicting mean blood flow in gray and white matter.

### Mediation analysis

Since eCRF and age were significantly correlated [*r*_(38)_ = −0.42, *p* < 0.01], an important question to address is whether eCRF mediates the age-related effects of mean CBF. Therefore, we performed the mediation analyses as described in the method section for the global gray matter mean flow and the parietal gray and white matter mean flow (i.e., the flow measures that showed relationships with both age and eCRF). For both global and parietal gray matter, we found that eCRF fully mediated the effects of age on CBF. However, no significant mediation by eCRF was evident for the effects of age on parietal white matter flow.

**Figure 5:**
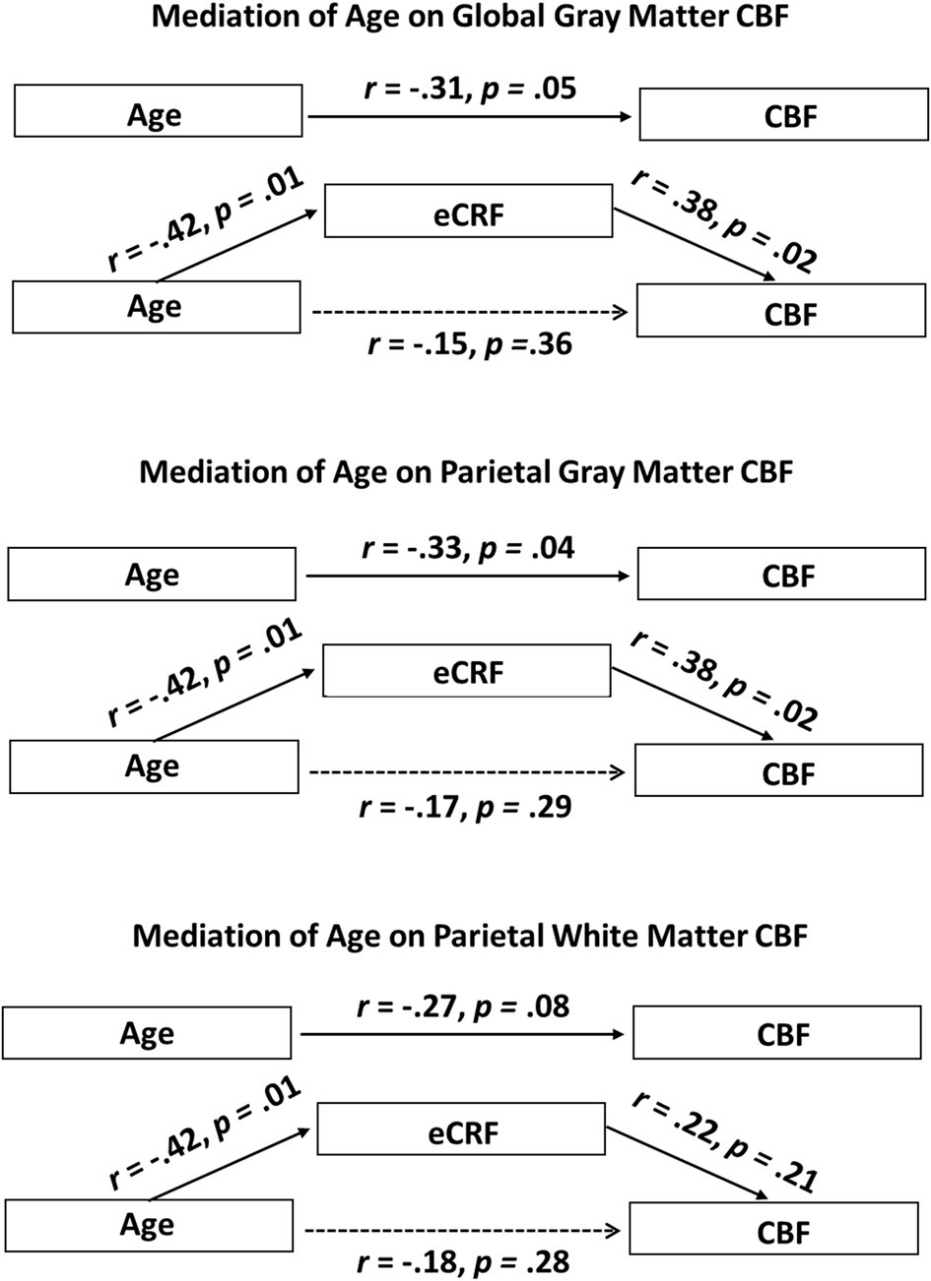
**Schematic representations of mediation analyses. Top panel:** Schematic representation of the results of the mediation analysis performed with the global gray matter CBF as the dependent variable. **Middle panel**: Schematic representation of the results of the mediation analysis performed with the parietal gray matter CBF as the dependent variable. **Bottom panel**: Schematic representation of the results of the mediation analysis performed with the parietal white matter CBF as the dependent variable. eCRF significantly mediates the effect of age on CBF in the global gray matter and parietal gray matter, but not in the parietal white matter.

Figure 5 (top panel) shows the path diagram of this mediation on global gray matter blood flow. Two-tailed significance tests were performed throughout the mediation analysis. Using the global measures of gray matter CBF, the regression of CBF on age, ignoring eCRF, was marginally significant [*b* = −0.35, *t*_(39)_ = −2.00, *p* = 0.05]. Furthermore, the regression of eCRF on age, also proved to be significant [*b* = −0.08, *t*_(39)_ = −2.89, *p* < 0.01]. Finally CBF was regressed on both age and eCRF at the same time. Critically, the unique effect of eCRF remained significant [*b* = −2.24, *t*_(39)_ = −2.39, *p* < 0.05] whereas the original effect of age completely disappeared [*b* = −0.17, *t*_(39)_ = −0.92, *p* = 0.36]. When using the bootstrap estimate, the mediation effect varied between −0.37 and −0.03 with 95% confidence. Because the 95% confidence interval did not include the 0 value, the result indicated a significant mediation effect.

A similar analysis was performed using the parietal measures of gray matter CBF (Figure 5, middle panel). The regression of these regional CBF measures on age was significant, [*b* = −0.38, *t*_(39)_ = −2.17, *p* < 0.05]. When CBF was regressed on age and eCRF at the same time, the effect of eCRF remained significant [*b* = 2.27, *t*_(39)_ = 2.39, *p* < 0.05], whereas the original effect of age went completely away [*b* = −0.20, *t*_(39)_ = −1.08, *p* = 0.29]. Similar to the global analysis, the bootstrapping procedure put the mediation effect between −0.45 and −0.01 at 95% confidence, indicating a significant mediation effect.

Finally, a third mediation analysis (Figure 5, bottom panel) was conducted using the CBF measures from the white matter located in the parietal region. The regression of these CBF measures on age was not significant using two-tailed significance tests [*b* = −0.22, *t*_(39)_ = −1.78, *p* = 0.08]. Here, when CBF was regressed on age and eCRF together, neither eCRF [*b* = 0.903, *t*_(39)_ = 1.29, *p* = 0.21] nor age [*b* = −0.15 *t*_(39)_ = −1.09, *p* = 0.28] remained predictive of CBF. Therefore, in the white matter, we did not observe any mediating effects of eCRF on age-related differences in CBF.

In our model, we conceptualize eCRF as the mediator of the well-known age effect on CBF. An alternative, perhaps less intuitive model, is that age acts a mediator of the eCRF effect on CBF. This result would be far less interesting and so further analysis is warranted to demonstrate that eCRF is the mediator of an age effect and not the other way around. Over the global gray matter, when testing age as the potential mediator of the effect of eCRF on CBF, the mediation effect varied between −0.34 and 1.43 with 95% confidence. Because the 95% confidence interval did include the 0 value, the result indicated a non-significant mediation effect. Likewise, in the parietal gray matter, the mediation effect varied between −0.27 and 1.39 with 95% confidence, and in the parietal white matter, the mediation effect varied between −0.22 and 1.10 with 95% confidence. Again, these results indicated that age was not a significant mediator of the effect of eCRF on CBF in the regions we analyzed. This result lends support for our original model, which conceptualized eCRF as the mediator.

### Analysis of eCRF components

Since we demonstrated that eCRF acted as a mediator of the age effects on CBF, it was of interest to understand how the individual components that were used to derive the eCRF score related to CBF. Because all of our prior analyses controlled for the effects of gender, we continued to partial out gender while reporting correlations for the sake of interpretability (Table 4). We observed the strongest correlation between the activity score component and global and parietal gray matter CBF. Interestingly, body mass index (BMI) was negatively correlated with frontal gray matter CBF, but not with the global CBF or parietal CBF. Both of these components had stronger correlations with CBF than age.

**Table 4:**
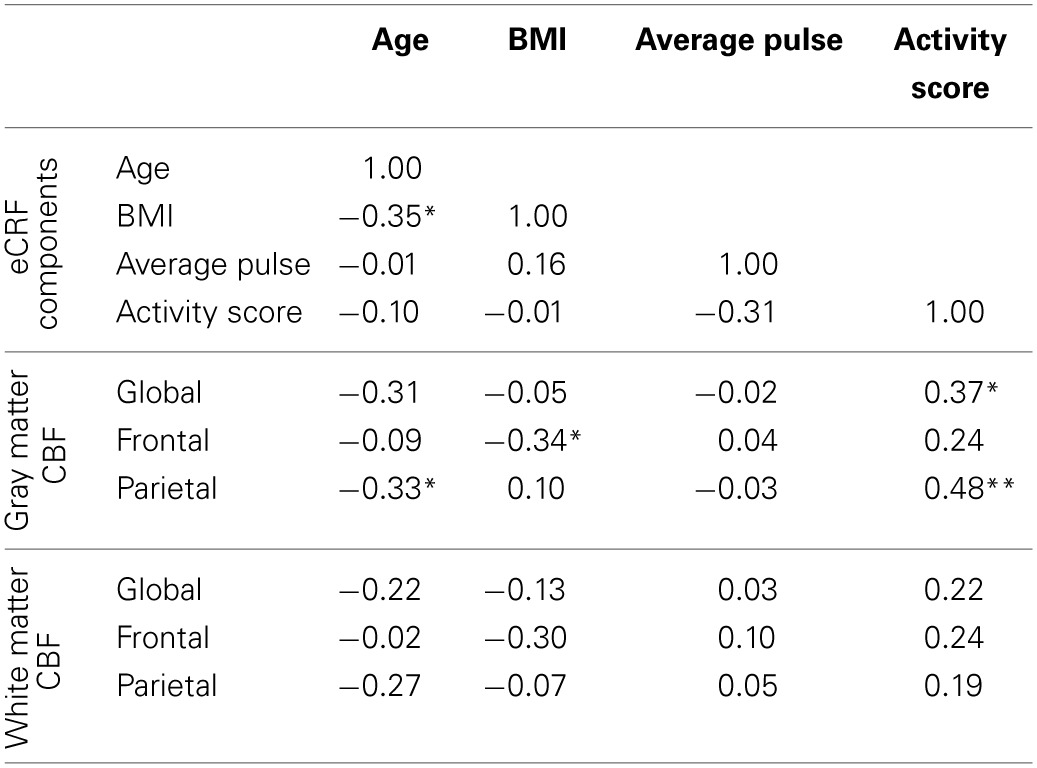
**Partial correlations between the individual components used to derive eCRF and measures of blood flow, while controlling for gender**.

Significance tests between groups (2-tailed): df = 38, ^*^p < 0.05, ^**^p < 0.01.

**Figure 6:**
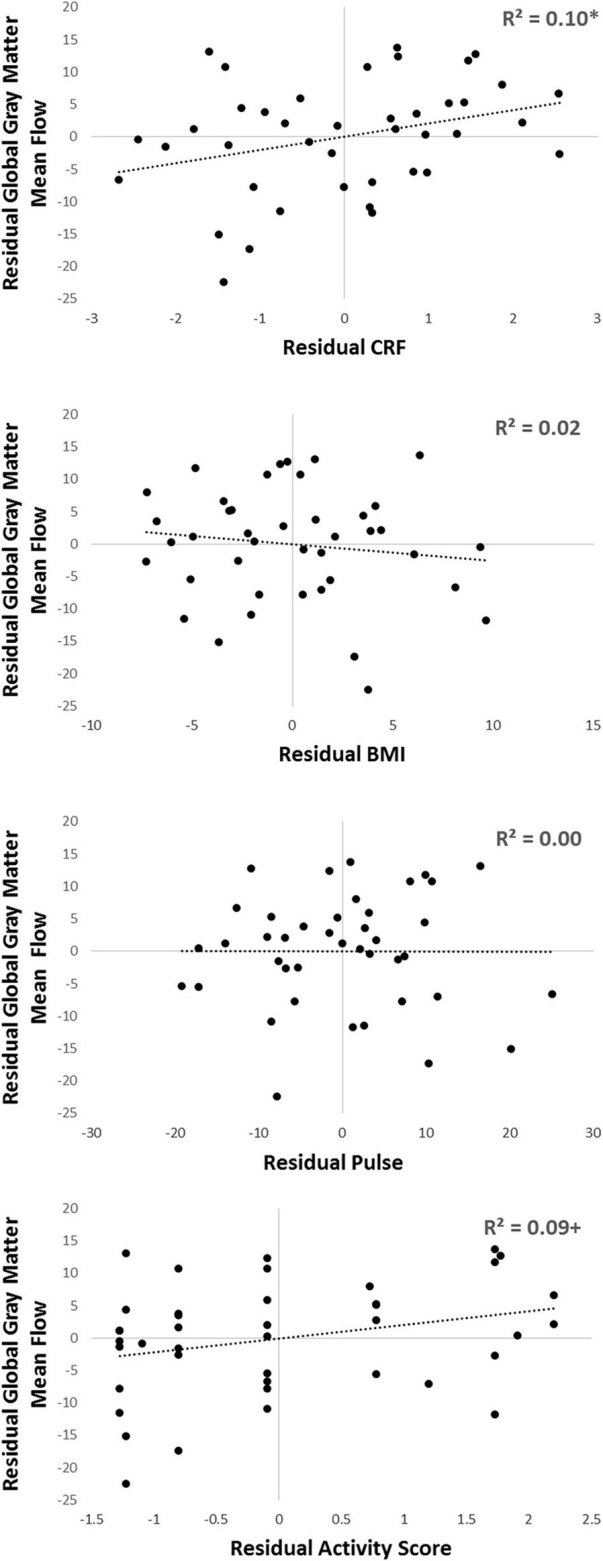
**Sensitivity analysis of the components comprising eCRF**. Participants were separated by gender, and then into older or younger groups by median split. Within each of those four groups, residuals of eCRF, BMI, pulse, activity score, and global gray matter CBF were calculated by subtracting the group mean from each of the observed scores. Shown here are the residuals of eCRF (top), and its individual components (BMI, pulse, and activity score; shown below) correlated with the residuals of CBF. ^+^*p* < 0.10, ^*^p < 0.05.

In addition, to further corroborate these findings, we carried out a sensitivity analysis, where we subdivided our sample first by gender, and then into younger and older groups within each gender. Within each of these four groups we calculated the mean eCRF and global gray matter CBF value, computed the residuals for each subject, and then computed correlations on the bases of these residual scores (thus effectively eliminating the confounds due to gender and age). The same procedure was also applied for the other three components of the eCRF score (BMI, pulse rate, and activity score; see Figure 6). In this stratified analysis, we hoped to further observe what aspects, if any, contributed to the relationship between eCRF and CBF when equating gender and age groups. We found that the residuals eCRF scores were still significantly correlated with the residual CBF scores [*r*_(39)_ = 0.28, *p* = 0.04]. Although neither the residuals of BMI nor those of pulse rate were correlated with the residuals of CBF, the residual activity scores were marginally significantly correlated with the residual CBF scores [*r*_(39)_ = 0.28, *p* = 0.07], which corresponds to our the analysis of the components of eCRF presented above.

### Analysis of anatomical volumes

In order to investigate whether or not localized CBF was predictive of other local brain measures, we analyzed the correlations between the volumes of the superior frontal and inferior parietal cortex and the regional measures of CBF (Table 5). These anatomical parcellations were chosen on the basis of their large size and likelihood to overlap with the frontal and parietal regional masks that were used to assess regional CBF in each individual subject. We observed a dissociation between the relationship of regional measures of CBF and differing regions of anatomy: frontal CBF predicted superior frontal brain volume but not inferior parietal volume, and parietal CBF marginally predicted inferior parietal volume but not superior frontal brain volume. These results suggest that local CBF may be related to variations in regional cortical volumes.

**Table 5:**
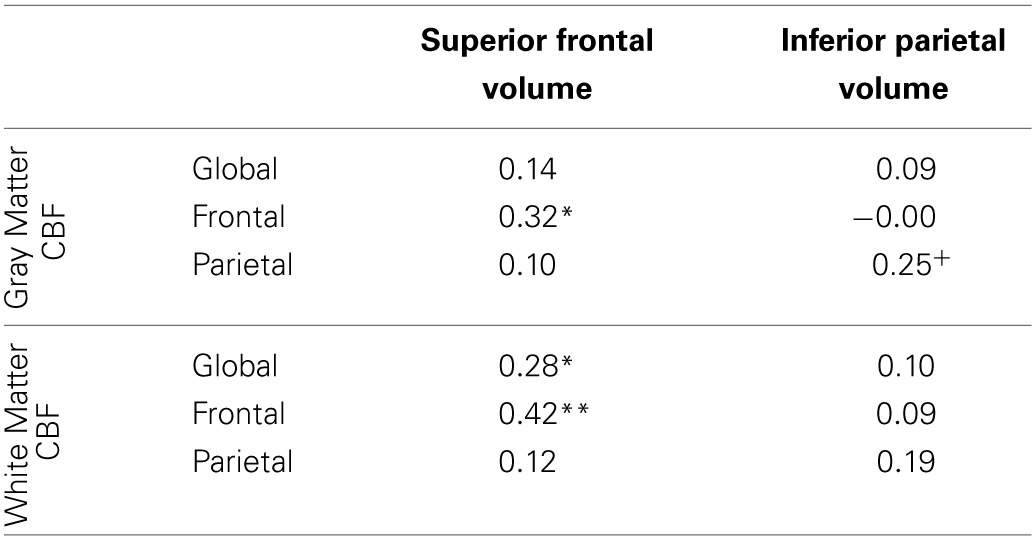
**Partial correlations between CBF and normalized superior frontal and inferior parietal anatomical volumes, controlling for gender**.

Significance tests between groups (1-tailed): df = 37, ^+^p < 0.10 ^*^p < 0.05, ^**p < 0.01.^

## Discussion

Our findings provide evidence that the declines in CBF that accompany aging are highly related to eCRF. CBF was negatively correlated with systolic blood pressure and pulse pressure, which provides support that CBF is, in fact, measuring perfusion rather than blood flow in the arteries. If CBF was measured in the arteries, we would have expected increases in blood pressure to be *positively* correlated with increased arterial blood flow. Furthermore, the fitness effects on CBF in the present analysis fully mediated the age effects on blood flow in the regions of the brain that showed a significant relationship between age and blood flow. Surprisingly, in frontal regions, no significant effect of aging on CBF was observed over the restricted age-range of our sample of older adults (aged 55–85). This is in contrast to anatomical studies that have reported reliable age-related changes in the frontal areas of the brain, when comparing younger adults (college age) to older adults (for a review of relevant work, see Raz, 2004). The limited age range of our study may account, at least in part, for this negative finding.

Our overall observation of a negative correlation between global CBF and age is consistent with previous reports using a variety of imaging techniques, including ASL (Parkes et al., 2004; Beason-Held et al., 2007; Ainslie et al., 2008). The findings reported here corroborate previous research indicating that improving eCRF may provide a route to stave off or even ameliorate the normal age-related declines in CBF, as well as the global cerebral atrophy that usually accompanies it (Ainslie et al., 2008). In fact, the mediation analyses suggest that a more direct relationship exists between eCRF and CBF than between age and CBF. If this is the case, then working to increase eCRF into old age may function as a potent method to preserve brain vascular health and stave off brain pathologies.

In our sample, it is clear that self-reported activity level seems to have a strong contribution to the overall impact of eCRF on CBF. This result is important for our interpretation, since there is an inherent “age” component in eCRF, and that component could have been explaining a large portion of the variance in CBF. The fact that activity level seems to play such a large role in explaining the impact of eCRF on CBF meshes well with other research that has demonstrated striking changes in the brains of older adults after cardiorespiratory exercise interventions (see Bherer et al., 2013 for a review). However, our analysis shows that the impact of eCRF may be more complex than simply reflecting the level of activity, since BMI shows a negative correlation with CBF only in the frontal brain areas that we examined. Complicating our interpretation, some of the variables that contribute to eCRF including pulse rate and BMI may have different implications depending on the time of life at which they are measured. For example, typically a higher resting pulse rate is considered to be a risk factor for cardiorespiratory disease (Fox et al., 2007). However, in older individuals a low resting pulse may also be indicative of heart disease (Ufberg and Clark, 2006). Likewise, BMI may be negatively correlated with fitness earlier in life, but as a sample increases in age, many individuals with high BMI will have expired and the correlation between BMI and fitness may reverse, as being underweight may be associated with more health problems (Yan et al., 2004). It is clear that eCRF is a complex measure, and the intriguing disparities in how the different components relate to flow in specific brain regions warrants future research. Specifically, given the correlations between many of the variables we analyzed, it is of particular importance to further investigate the relationship between eCRF and CBF in aging adults. Longitudinal studies and interventions that improve eCRF will help to further elucidate the impact of fitness on the age-related declines in CBF.

Although our analysis of the white matter revealed similar patterns to the gray matter analysis, CBF in the white matter may not be functionally equivalent to CBF in the gray matter. Parkes et al. (2004) reported a positive trending correlation (*r* = 0.34; *p* = 0.10) between age and white matter CBF. More recently, Lu et al. (2011) also showed that in certain areas of the white matter CBF increased with age. An age-related increase in white matter CBF alongside of decreases in gray matter CBF may appear to be counterintuitive. A potential explanation was proposed by Aslan et al. (2011). In their study, the authors found an inverse correlation between the anisotropy of water diffusion and the blood flow along white matter fiber tracts. This suggests that the deterioration of white matter integrity is positively related to a local rise in CBF. This inverse relationship is most likely due to the structure of the myelinated axons in the white matter. Demyelination of axons could lead to an increased need for greater ion flux and energy for depolarization, since nodes of Ranvier normally minimize this need. In addition, it may also reduce the ability of the structure of the white matter to offset the pressure of perfusion. Both of these factors may lead to increased blood flow (Aslan et al., 2011). Overall, this study points out that the blood flow in the white matter may not be completely determined by neurovascular coupling in the same way that it is in the gray matter. The measures of white matter blood flow we reported likely reflect a combination of blood flow downstream of the gray matter along with the effects of any age-related white matter degradation. Since these two age-related factors impact blood flow in different directions, it is likely that our measures of CBF in the white matter are much less sensitive to age-related changes, and much more difficult to interpret.

The positive impact of fitness on CBF during healthy aging may work in a number of ways, mostly involving improvements in the cardiovascular system at different levels. Maintaining a higher level of fitness has been shown to reduce many cardiovascular risk factors, such as hypertension and diabetes, which may interact with the brain in complex ways (Clarkson et al., 1999; Jennings and Zanstra, 2009). Chronic hypertension has been shown to correlate with long term structural changes in small cerebral vessels (Baumbach and Heistad, 1989), cerebral microbleeds (Wang et al., 2009), brain atrophy (Salerno et al., 1992), and even the permeability of the blood-brain barrier (Yang et al., 1991). Although these examples reflect clear disease states, preclinical physiological changes, such as plaque deposition and insulin resistance, emerge over time. It is possible that accruing these types of changes over time may be involved in age-related declines in brain cognitive function, and that reversing or slowing this process would lead to protection against related declines. It has also been shown that habitual physical activity is associated with greater endothelial health, including increased endothelium-dependent vasodilation and availability of nitric oxide, which may serve to reduce oxidative stress (Taddei et al., 2000). Lavi et al. (2006) have demonstrated that endothelial dysfunction disrupts vascular reactivity to CO_2_, and may disrupt CBF regulation that is dependent on CO_2_ levels. This is especially interesting given the age-related impairments in vasodilation to hypercapnia seen by Safonova et al. (2004). Finally, certain chemical factors have also been shown to be upregulated with exercise, and have been implicated in some of the neuroprotective effects of fitness (Kramer et al., 2006). Most research has revolved around brain-derived neurotrophic factor (BDNF) and insulin-like growth factor 1 (IGF-1), which may improve angiogenesis and neurogenesis (Black et al., 1990; Neeper et al., 1995; Carro et al., 2001; Garza et al., 2004; Kramer et al., 2006). Recent research has also shown correlations between cellular viability (as indexed by N-acetylaspartate) and eCRF (Erickson et al., 2012). All these mechanisms may lead either directly or indirectly to increased CBF, and each of them may also lead to improved overall brain health and cognitive function. It has been known for some time that aerobic exercise interventions, which serve to improve eCRF, show benefits in both physical and cognitive health in aging subjects (Colcombe and Kramer, 2003; Colcombe et al., 2006). However, what the current study illustrates is how much of the normal physiological declines present in healthy aging is related to decreased fitness. In fact, our findings indicate that a substantial portion of the variance in brain health that was previously attributed to “aging” in general is mediated by declines in eCRF.

The finding that localized measures of CBF are related to localized measures of brain volume supports the effectiveness of ASL as a tool to investigate localized perfusion in the brain, as well as the importance of investigating differences between brain regions. In this particular study, the total global measures of CBF were split up into regions to assess whether CBF differences in these more specific areas were related to local differences in brain volume. In the future, it will be critical to examine even smaller regions to determine how sensitive this measure of CBF truly is to local brain health, beyond global effects. Given the correlative nature of studying CBF in normal aging humans, it is impossible to determine whether changes in volume lead to changes in CBF, or if CBF can be an important mediator of future changes in brain volume. Further longitudinal research may help elucidate how current regional CBF is involved in predicting subsequent regional changes in brain volume or other measures of brain health.

### Methodological considerations

There is an inherent difficulty in studying healthy aging participants. In order to volunteer for and attend our studies, older adults must necessarily have some degree of physical independence and mobility. Many people who may not be fit or genetically protected against vascular disease may die or be incapable of participation before they are old enough to be included in the study. This problem manifests itself in a possible underestimation of the impact of fitness on CBF. Furthermore, because the average life expectancy of females is greater than the life expectancy of males, sampling only from the oldest group of subjects may allow for an oversampling of extra fit males compared to females. Given that baseline CBF may already differ between men and women (Kastrup et al., 1999), it is especially important to control for gender differences when analyzing CBF.

Another potential confound arises while studying CBF with fMRI. Individual voxels likely contain contributions of both some white and gray matter, which could lead to different flow values in separate voxels labeled as gray matter, based solely on differential contributions of white and gray matter. These partial volume effects could lead to misinterpretations of CBF results. However, blood pressure was negatively correlated with CBF, demonstrating that there is a relationship between CBF and at least one physiological measure that is independent from any partial volume effects. This lends support to the claim that the results reported here are not simply artifacts of partial volume effects.

## Summary and conclusions

The main findings of our study indicated that eCRF is strongly correlated with CBF explored by our 6 slices in the brain, and that the effects of age on CBF are significantly mediated by eCRF. It should be emphasized that this effect is observed in an otherwise healthy group of older adults, and refers to benefits in “normal” aging as opposed to benefits for protection against pathology. By maintaining higher eCRF, it appears possible to substantially impact the well-established age-related declines in CBF.

### Conflict of interest statement

The authors declare that the research was conducted in the absence of any commercial or financial relationships that could be construed as a potential conflict of interest.

## Footnotes

^1^About 30% (*N* = 12) of our sample show blood pressure values that are consistent with isolated systolic hypertension (systolic >140, diastolic <90). Isolated systolic hypertension appears to be a very common feature in older adults (Basile, 2002). Therefore, we felt that eliminating subjects with this particular syndrome could bias our sample and decided to keep these participants in the study.

^2^Although it is true that intracranial size is related to gender, intra-individual differences in intracranial volume represent more than only gender, so it is important to control for both. Further support for this comes from recent evidence showing that intracranial volume measurements themselves may be biased by gender (Nordenskjöld et al., 2013). Since finding partial correlations with gray-matter volume requires that gender be removed from both, we believe that removing gender after correcting for intracranial volumes was justified.
